# Supplementary material for: Analysis of PPARγ Signaling Activity in Psoriasis
Source: Int J Mol Sci. 2021 Aug 10;22(16):8603. doi: 10.3390/ijms22168603 (PMC8395241; doi:10.3390/ijms22168603)
Supplement: Supplementary file 1 [file ijms-22-08603-s001.zip › Supplemental materials_Analysis of PPARg signaling activity in psoriasis/Pathway models/Models images and html files/PPARG inhibits phenotypes assotiated with psoriasis/134632.html]

diabetes mellitus


# Disease diabetes mellitus

|  |  |
| --- | --- |
| URN | urn:agi-meshdis:Diabetes%20Mellitus |
| Total Entities | 13 |
| Connectivity | 19357 |
| Name | diabetes mellitus |

---

|  |  |
| --- | --- |
| ChildConcepts | type 1 diabetes |
|  | diabetic complication |
|  | maternally inherited diabetes and deafness |
|  | cystic fibrosis-related diabetes |
|  | lipoatrophic diabetes |
|  | type 2 diabetes |
|  | prediabetes |
|  | gestational diabetes |
|  | insulin-resistant diabetes |
|  | Wolfram syndrome |
|  | macrovascular complication |
|  | neonatal diabetes mellitus |
|  | muscular atrophy with ataxia, retinitis pigmentosa, and diabetes mellitus |

---

|  |  |
| --- | --- |
| Pathway | Proteins with Altered Expression in Aging |
|  | Protein Oxidation and Nitration Products as Disease Biomarkers |
|  | PASK Senses and Regulates Glucose Homeostasis |
|  | GLIS3 Targets in Thyroid Dysgenesis (Hypothesis) |
|  | CDKN2B-AS1 in Epigenetic Regulation of Glaucoma |
|  | Ritonavir Induced Diabetes |
|  | PPARG inhibits phenotypes assotiated with psoriasis |
|  | efects selected |
|  | Neighbors of diabetes mellitus |

---

|  |  |
| --- | --- |
| MedScan ID | 9010191 |

---

|  |  |
| --- | --- |
| Alias | Diabetes Mellitus |
|  | diabets mellitus |
|  | diabetic |
|  | Diabetes |
|  | diabets |
|  | Unspecified Diabetes Mellitus |

---

|  |  |
| --- | --- |
| MeSH ID | D003920 |
|  | M0006148 |

---

|  |  |
| --- | --- |
| MeSH Heading | Diabetes Mellitus |

---
